# The Mechanism of Omicron Variant‐Associated Cardiac Injury in Rhesus Macaques Was Revealed by Proteomic and Phosphoproteomic Analyses

**DOI:** 10.1002/mco2.70266

**Published:** 2025-06-19

**Authors:** Tao Ding, Ya‐Nan Zhou, Jiang‐Feng Liu, Shuai‐Yao Lu, Jun‐Tao Yang

**Affiliations:** ^1^ State Key Laboratory of Common Mechanism Research for Major Diseases Department of Biochemistry and Molecular Biology Institute of Basic Medical Sciences Chinese Academy of Medical Sciences School of Basic Medicine Peking Union Medical College Beijing China; ^2^ National Kunming High‐level Biosafety Primate Research Center Institute of Medical Biology Chinese Academy of Medical Sciences and Peking Union Medical School Kunming China

1

Dear Editor,

The Omicron variant of SARS‐CoV‐2 quickly surpassed the previously dominant Delta variant. Compared to previous variants of concern (VOCs), it was highly mutated, high reinfection, highly transmissible, and rapidly spreading strain. The Omicron variant infected cells that depended on angiotensin‐converting enzyme 2 (ACE2). The increased expression of ACE2 in cardiomyocytes made cardiac tissue more susceptible to Omicron variant infection. Myocardial injury was a common comorbidity in patients with SARS‐CoV‐2 infection and portended a poor prognosis. Consequently, studying myocardial injury caused by the Omicron variant is urgent compared to previous VOCs. To determine how the Omicron variant hijacks protein signaling in different cardiac regions, the cardiac samples were harvested for proteomic and phosphoproteomic analyses (Figure 1A). SARS‐CoV‐2 RNA was detected in the heart, further suggesting that SARS‐CoV‐2 can infect the heart tissue directly (Figure 1A). H&E staining revealed myocardial hemorrhage, infiltration of inflammatory cells, and atrophy of myocytes in the infected hearts, and Masson staining revealed significant myocardial fibrosis in the infected hearts, which are characteristic features of human COVID‐19 cardiac injury [1] (Figure 1A).

**FIGURE 1:**
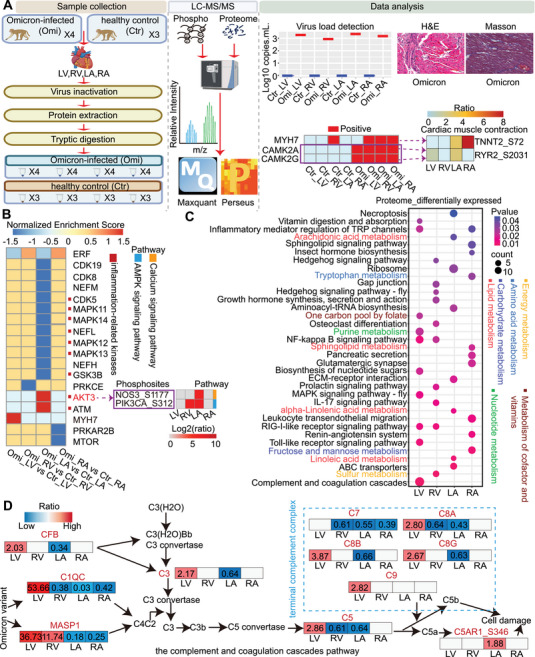
Global proteomic and phosphoproteomic profiling of the four cardiac regions of rhesus macaques infected with the Omicron variant. (A) Brief workflow of the study. Seven rhesus macaques were divided into healthy control and Omicron‐infected groups. The four regions of their hearts (LV, RV, LA, and RA) were extracted for viral load detection, morphological analyses, protein extraction, and LC‐MS/MS detection. After the MaxQuant‐based database search and Perseus‐based data rearrangement, proteomic and phosphoproteomic data were processed for bioinformatics analyses and data mining. LV: left ventricle, RV: right ventricle, LA: left atrium, RA: right atrium, Phospho: phosphoproteome, Ratio = mean of Omicron‐infected group/mean of healthy control group, ratio>1.5: upregulated phosphosites. (B) Left heatmap of predicted kinases that may have significantly altered activity following infection with the Omicron variant. Kinase activity is represented by NES values. The activation threshold for kinases is defined by an NES value greater than 0, while the inhibition threshold is characterized by an NES value less than 0. Right heatmap representing the expression of upregulated phosphosites in the AMPK signaling pathway and calcium signaling pathway in the four cardiac regions, ratio > 1.5 were considered upregulated phosphosites. NES: Normalized Enrichment Score. (C) Functional enrichment analyses for differentially expressed proteins in the four cardiac regions. (D) Map of proteins in the complement and coagulation cascades pathway in the four cardiac regions of rhesus macaques following infection with the Omicron variant. The blue‐to‐red color gradient represents the ratio of average protein abundance in the Omicron variant versus the healthy control group in each region.

Previous studies conducted phosphoproteomic analysis on cardiomyocytes infected with SARS‐CoV‐2 [2], finding significant changes in the phosphoproteomes, consistent with the central role of phosphorylation in cellular signal transduction. Thus, we performed the global phosphoproteomic analysis and revealed that differentially expressed phosphoproteins in the four regions were concentrated in the cardiac muscle contraction pathway (Figure 1A), which may be associated with COVID‐19‐related arrhythmias [3]. To explore the biological significance, we performed kinase prediction using GPS 5.0 and GSEA. We highlighted key kinases, including CAMK2A and CAMK2G in modulating the phosphosites RYR2_S2031. Furthermore, MYH7 may be involved in the phosphorylation regulation of TNNT2_S72. Although the kinase function of MYH7 required further validation, our findings suggested its potential biological significance (Figure 1A). CAMK2A and CAMK2G exhibited significant activation in the four cardiac regions following Omicron variant infection (Figure 1A). CAMK2 increased RYR2‐mediated sarcoplasmic reticulum (SR) calcium leakage by phosphorylating specific RYR2 residues, which trigger cardiac arrhythmias. Thus, the upregulation of phosphorylation at RYR2_S2031 may signal arrhythmia risk following Omicron variant infection. KN‐93, a CAMK2 inhibitor, has previously been used in the treatment of ventricular arrhythmias [4]. The results suggested that CAMK2A and CAMK2G could be potential targets for treating arrhythmias caused by Omicron variant infections.

We plotted the normalized enrichment score (NES) values of kinases to compare kinase activity patterns in the four cardiac regions (Figure 1B). Our analysis revealed that kinase activity significantly altered in the left atrium (LA), with many closely linked to inflammation. Notably, the LA had the highest viral load concentration (Figure 1A). These findings further heightened the susceptibility of the LA to viral infection and inflammatory storms in COVID‐19 patients [1], possibly because the LA was connected to the pulmonary vein, which was more susceptible to the virus that infected the lung and its inflammatory storm. These suggested a need for enhanced monitoring of LA‐related inflammatory kinase activity in clinical COVID‐19 patients. Given that kinases play a crucial role in modulating myocardial conduction, we specifically focused on the calcium signaling pathway and AMPK signaling pathway in the LA. Interestingly, we discovered that the positive kinases AKT3 targeted the phosphosites NOS3_S1177 in the calcium signaling pathway and PIK3CA_S312 in the AMPK signaling pathway, and they were upregulated in the LA (Figure 1B). These findings elucidated the regulatory mechanisms of AKT3 within conduction system diseases. Future studies could explore the potential of intervening in the AKT3 signaling pathway to protect cardiac function.

Additionally, proteomic analysis revealed significant changes in the overall metabolism, and the metabolic pathways in multiple organs of COVID‐19 patients also undergo significant changes, further heightened the importance of metabolic pathways in the pathophysiology of COVID‐19. Metabolic pathways varied in the four cardiac regions (Figure 1C), with lipid metabolism in the LA, nucleotide metabolism in the LV, carbohydrate metabolism in the RA, and energy metabolism in the RV, suggesting the Omicron variant may altered metabolic pathways of the host cells during replication. We identified molecules in the metabolic pathways to elucidate the specific functional changes. For instance, HK2 in the carbohydrate metabolism pathway was upregulated in three regions other than the LA, facilitating autophagy to protect cardiomyocytes. Further research can focus on targeting HK2‐related carbohydrate metabolism to prevent myocardial damage.

Left ventricular diastolic dysfunction was observed in hospitalized COVID‐19 patients. Then our research revealed a significant upregulation of the complement and coagulation systems in the left ventricle (LV) (Figure 1D), possibly because of the left ventricle's diastolic dysfunction, key for blood pumping, which may cause blood stasis and activate complement and coagulation pathways. Notably, terminal complement complex (TCC) was significantly upregulated in the LV, linking its activation to microthrombus formation. The elevated levels of CF1 and F12 in the LV, particularly the contribution of F12 to intensify the formation of microthrombi. These findings could explain the cardiac injury and coagulation abnormalities observed in COVID‐19 patients. In addition, proteins such as MASP1, C3, CFB, C5, C7, C8A, C8B, C8G, and C9, were also identified in the serum of patients with Long Covid [5], echoing the sustained activation of the complement system in long‐COVID‐19 patients. In summary, our study provides new insights into the mechanisms of myocardial injury following Omicron variant infection, highlighting the importance of preventing excessive complement activation and increased coagulation risks in the LV.

This study had several shortcomings that warrant consideration. Firstly, the sample size was limited due to the scarcity of rhesus macaque samples, resource constraints, and ethical considerations. Secondly, we did not account for gender factors. Future research will expand the sample size and increase gender considerations. Finally, studies utilized cardiac samples from rhesus macaques, clinical tissues are the preferred samples for molecular and mechanistic studies, but the availability of COVID‐19 clinical tissues is limited. Therefore, developing animal models, particularly nonhuman primate models, is crucial for researching the Omicron variant.

In summary, this study focused on the Omicron variant, revealing that CAMK2A and CAMK2G could be potential targets for treating arrhythmias caused by Omicron variant infections. Moreover, the Omicron variant caused different metabolic changes in four regions and affected conduction system diseases through the positive kinases AKT3 in the LA. Additionally, the complement and coagulation systems exhibited significant upregulation in the LV. The study provides the proteomic and phosphoproteomic profiles of the different cardiac regions following the Omicron variant infection, guiding future clinical treatments for cardiovascular diseases associated with the Omicron variant.

## Author Contributions

J.T.Y., S.Y.L., and J.F.L. designed the experiments. Y.N.Z. performed the operation of viral infection in animal models and detected the viral load. T.D. wrote and revised the manuscript. T.D., Z.Y.Z., Y.W., X.T.Z., X.Y.T., W.J.P., C.M.S., and Q.C.W. analyzed the proteomics and phosphoproteomics data. All authors have read and approved the final manuscript.

## Ethics Statement

Ethical approval for all animal‐related procedures in this study was obtained from the Institutional Animal Care and Use Committee of the Institute of Medical Biology, Chinese Academy of Medical Science (approval number: DWSP202002 001).

## Conflicts of Interest

The authors declare they have no conflicts of interest.

## Supporting information




Supporting Information

## Data Availability

All proteomics and phosphoproteomic raw data have been deposited to the ProteomeXchange Consortium with the dataset identifier PXD051632. URL: （https://www.iprox.cn/page/PSV023.html;?url = 1732494491431wZMy ）. Password: EPMp.
