# Supplementary material for: The Mechanism of Omicron Variant‐Associated Cardiac Injury in Rhesus Macaques Was Revealed by Proteomic and Phosphoproteomic Analyses
Source: MedComm (2020). 2025 Jun 19;6(7):e70266. doi: 10.1002/mco2.70266 (PMC12179395; doi:10.1002/mco2.70266)
Supplement: Supplementary file 1 — Supporting Information [file MCO2-6-e70266-s001.docx]

**Supplementary Materials and Methods for**

**The mechanism of** **Omicron variant-associated** **cardiac injury in** **rhesus macaques was revealed by proteomic and phosphoproteomic analyses**

Tao Ding^1 #^, Ya-Nan Zhou^2 #^, Jiang-Feng Liu^1 *^, Shuai-Yao Lu^2 *^, Jun-Tao Yang^1 *^

^1^State Key Laboratory of Common Mechanism Research for Major Diseases, Department of Biochemistry and Molecular Biology, Institute of Basic Medical Sciences Chinese Academy of Medical Sciences, School of Basic Medicine Peking Union Medical College, Beijing, China.

^2^National Kunming High-level Biosafety Primate Research Center, Institute of Medical Biology, Chinese Academy of Medical Sciences and Peking Union Medical School, Kunming, China.

#Co-first author: Contribute equally to this work.

*Corresponding authors: Jun-Tao Yang: yangjt@pumc.edu.cn;

Shuai-Yao Lu: lushuaiyao-km@163.com;

Jiang-Feng Liu: [ljf@pumc.edu.cn](mailto:ljf@pumc.edu.cn)

**Methods and Materials**

**Ethics and biosafety statement**

All rhesus macaques were sourced from the Institute of Medical Biology, Chinese Academy of Medical Sciences, and were in good health, having not been involved in any other experimental procedures. The study was approved by the Institutional Animal Care and Use Committee of the Institute of Medical Biology, Chinese Academy of Medical Sciences (ethics number: DWSP202101001). All animals had free access to water and a standard diet and were housed under a 12-hour light-dark cycle (temperature: 20-23℃, humidity: 45%-65%). All procedures followed the guidelines for the National Care and Use of Animals, approved by the National Animal Research Authority and the ABSL-3 facility of the National Kunming High-level Biosafety Primate Research Center, Yunnan, China.

**Virus amplification and identification**

Omicron BA.1 (B.1.1.529 CCPM-B-V- 049-2112-18) was obtained from the National Kunming High-level Biosafety Primate Research Center. Viruses were amplified in Vero cells, purified, and concentrated with an ultrafilter system with a 300-kDa module (Millipore, US). The SARS-CoV-2 Omicron variant was confirmed via reverse-transcription polymerase chain reaction (RT‒PCR), sequencing, and transmission electronic microscopy and titrated via a plaque assay (10^7^ plaque-forming units [PFU]/mL).

**Animal experimental procedures**

Seven rhesus macaques were used in this study (six females and one male, aged 2-11 years). The animals were divided into two groups: the virus infection group (n=4), which received intranasal inoculation with the SARS-CoV-2 Omicron variant, and the blank control group (n=3), which received no treatment. Before viral inoculation, the animals were anesthetized with ketamine (6 mg/kg). Each animal in the virus infection group was infected with 1 mL of 1 × 10^6^ PFU of the SARS-CoV-2 Omicron variant (500 µL intranasally and intratracheally). All rhesus macaques met the criteria for inclusion in the subsequent analysis. The animals were sacrificed at 7 days post-infection (dpi), which is a critical time point when the viral load in the lungs begins to decline after reaching a peak, characterized by severe pathological damage, and typical COVID-19 features. Pulmonary changes affect systemic organs, including the heart. Thus, 7 dpi is ideal for studying cardiac pathology. Additionally, tissue samples were harvested for viral load quantification, histopathological examination, and proteomic and phosphoproteomic analyses.

**Morphological analysis**

Heart tissue samples from four anatomical regions from the SARS-CoV-2 Omicron variant-infected group and the control group were harvested and fixed in 10% neutral-buffered formalin for 3-7 days. Formalin-fixed paraffin-embedded tissues were cut into 5 μm sections for H&E staining and histopathological analysis.

**Viral RNA extraction and quantification of the viral RNA genome**

A TRIzol suspension of 400 μL of swab or 100 mg of tissue sample from each animal was used for RNA extraction using a Direct-zol RNA Miniprep Extraction Kit (Zymo Research, catalog no. R2052) according to the manufacturer’s instructions. Swab samples soaked in TRIzol solution were vortexed, and then the swabs were removed. The TRIzol suspension obtained from the swabs (400 μL) was used to extract the RNA, which was washed with 50 μL of DNase/RNase-free water to elute the RNA, followed by storage at -80 °C. For tissue homogenization, 1 mL of TRIzol was added to 100 mg of tissue. The supernatant (400 μL) was centrifuged to extract the RNA template, which was washed with 50 μL of DNase/RNase-free water to elute the RNA and stored at -80 °C. Real-time PCR (RT‒PCR) was used to quantify the viral genome using TaqMan Fast Virus 1-Step Master Mix (Thermo Fisher Scientific), and SARS-CoV-2 RNA was used for the standard curve. RT‒PCR was performed on a CFX384 Touch Real-Time PCR Detection System (Bio-Rad).

**Sample preparation and LC-MS/MS analysis**

Approximately 150 mg of tissue sample from each animal was used for protein extraction. For digestion, trypsin was added at a 1:50 trypsin-to-protein mass ratio overnight. Finally, the peptides were desalted and stored until subsequent peptide fractionation. The peptide samples for proteome analysis were fractionated by high-pH reverse-phase HPLC using an Agilent 300 Extend C18 column (5 mm particles, 4.6 mm ID, 250 mm length). For phosphopeptide enrichment, peptide mixtures were first incubated with a Fe-IMAC microsphere suspension with vibration in loading buffer (50% acetonitrile/0.5% acetic acid). The supernatant containing phosphopeptides was collected and lyophilized for LC-MS/MS analysis. An Orbitrap Exploris^TM^ 480 mass spectrometer (Thermo Fisher Scientific) equipped with an EASY-nLC 1200 UPLC system was used for tandem mass spectrometry (MS/MS) analysis. A binary buffer system consisting of buffer A (0.1% formic acid in 2% acetonitrile) and buffer B (0.1% formic acid in 90% acetonitrile) was used for peptide separation. An electrospray voltage of 2.3 kV was applied. The intact peptides were detected in the orbitrap at a resolution of 60,000. Peptides were then selected for MS/MS with NCE 27, and the fragments were detected in the orbitrap at a resolution of 15,000. A data-dependent procedure that alternated between one MS scan and 25 MS/MS scans with 20 s dynamic exclusion was used. For proteome analysis, the automatic gain control (AGC) was set at 100%, with an intensity threshold of 5E4 and a maximum injection time set to Auto. For phosphoproteome analysis, the maximum injection time is set at 100 ms.

**Database search and bioinformatics analysis**

The resulting MS/MS raw files were processed using the MaxQuant search engine (v1.6.15.0) against the *Macaca mulatta* database (UniProt, Macaca_mulatta_9544_PR_20230529.fasta (44389 sequences)) concatenated with the reverse decoy database. Trypsin/P was specified as a cleavage enzyme, allowing up to 2 missing cleavages. Carbamidomethyl (C) was specified as a fixed modification. Oxidation (M), acetylation (protein N-terminal), deamidation (NQ), and phosphorylation (STY) were specified as variable modifications. The FDR was adjusted to < 1% and the minimum score for modified peptides was set to > 40. The minimum peptide length was set at 7. For the quantification method, matching between runs was enabled. All the other parameters in MaxQuant were set to default values. MaxQuant-derived data were submitted to Perseus for subsequent processing. First, proteins matched to potential contaminants and reverse databases were excluded. Then, only phosphosites whose localization probabilities were>0.75 were retained. Proteins/phosphosites that existed in>50% of the samples in the control or virus-infected group were considered quantifiable. Normalization and imputation were performed using the “Divide” (by median of column) and “Replace missing values from normal distribution” functions in Perseus. As with previous studies, we did not normalize the intensity of phosphosites to the corresponding protein levels (protein abundance normalization).

**Statistical analyses**

Two independent sample t-tests for paired comparisons were used to determine the differentially expressed proteins/phosphosites and the cutoff was set at p < 0.05. The fold change (FC) was calculated by dividing the mean value of the virus-infected group by that of the control group (FC=mean of virus-infected group/mean of the control group). Proteins/phosphosites with p < 0.05 and FC > 1.5 were considered upregulated, and those with p < 0.05 and FC < 0.67 were considered downregulated. The KEGG database was used (Fisher’s exact test; Benjamini and Hochberg method for FDR correction). Pathways with a P value < 0.05 (Fisher’s exact test) were defined as enriched. Kinase prediction was based on kinase-substrate relationships recorded by GPS5.0 that utilizes the theory that short linear motifs around phosphosites are specifically recognized by specific kinases. The threshold parameter was set to ‘medium’. The interaction parameter was set to ‘Exp./String’ to filter potentially false-positive hits. Kinase activity prediction was based on the gene set enrichment analysis (GSEA) algorithm, as the intensity of phosphosites could reflect the activity of corresponding kinases. The .rnk file was set to the log-transformed FC of phosphosites, and the .gmt file was set to the corresponding kinase-substrate relationships. The generated NES of the enrichment results was regarded as the kinase activity score. The cutoff of the predicted activated/ inhibited kinases was set to NES >0/ NES <0.
